# Chitosan-based biomaterial delivery strategies for hepatocellular carcinoma

**DOI:** 10.3389/fphar.2024.1446030

**Published:** 2024-08-05

**Authors:** Xianling Wang, Yan Yang, Shuang Zhao, Di Wu, Le Li, Zhifeng Zhao

**Affiliations:** ^1^ Department of Gastroenterology, The Fourth Affiliated Hospital of China Medical University, Shenyang, China; ^2^ Endoscopy Center, The Fourth Affiliated Hospital of China Medical University, Shenyang, China; ^3^ First Digestive Endoscopy Department, The Fourth Affiliated Hospital of China Medical University, Shenyang, China

**Keywords:** biomaterials, chitosan, hepatocellular carcinoma, apoptosis, antioxidant, antiinflammatory, angiogenesis inhibition, extracellular matrix remodeling

## Abstract

**Background:**

Hepatocellular carcinoma accounts for 80% of primary liver cancers, is the most common primary liver malignancy. Hepatocellular carcinoma is the third leading cause of tumor-related deaths worldwide, with a 5-year survival rate of approximately 18%. Chemotherapy, although commonly used for hepatocellular carcinoma treatment, is limited by systemic toxicity and drug resistance. Improving targeted delivery of chemotherapy drugs to tumor cells without causing systemic side effects is a current research focus. Chitosan, a biopolymer derived from chitin, possesses good biocompatibility and biodegradability, making it suitable for drug delivery. Enhanced chitosan formulations retain the anti-tumor properties while improving stability. Chitosan-based biomaterials promote hepatocellular carcinoma apoptosis, exhibit antioxidant and anti-inflammatory effects, inhibit tumor angiogenesis, and improve extracellular matrix remodeling for enhanced anti-tumor therapy.

**Methods:**

We summarized published experimental papers by querying them.

**Results and Conclusions:**

This review discusses the physicochemical properties of chitosan, its application in hepatocellular carcinoma treatment, and the challenges faced by chitosan-based biomaterials.

## 1 Introduction

Hepatocellular carcinoma (HCC) is the predominant form of primary liver cancer, accounting for over 80% of liver cancer cases (Valderrama-Treviño et al., 2017). HCC is closely associated with hepatitis B, hepatitis C, alcoholic liver disease, and fatty liver disease (Kulik and El-Serag, 2019). The incidence of HCC is higher in men, predominantly due to higher rates of alcohol consumption (Villanueva, 2019). Globally, the incidence of HCC is steadily increasing, projecting over 1 million new cases per year by 2025 (Rahib et al., 2014). HCC is often asymptomatic in its early stages, leading to diagnosis at advanced stages with limited treatment options, poor prognosis, and short survival times (Fan et al., 2023). HCC ranks as the third leading cause of cancer-related deaths globally, with a 5-year survival rate of approximately 18% (Siegel et al., 2022). In the United States, the average 5-year survival rate for HCC patients is 19.6% (Chidambaranathan-Reghupaty et al., 2021).

Patients with HCC have various treatment options, including liver transplantation, surgical resection, percutaneous ablation, radiotherapy, chemotherapy, and targeted or systemic therapy (Vogel et al., 2022). Surgical resection is the preferred and most effective treatment for early-stage HCC (Sugawara and Hibi, 2021; Duong et al., 2022). However, it is unsuitable for unresectable and metastatic HCC. Chemotherapy is commonly used but often accompanied by systemic side effects (Bhatt and Wu, 2023). Advanced HCC patients who cannot undergo surgery face challenges due to tumor lesions and the development of treatment resistance (Zhao et al., 2022a; Zhao et al., 2022b). Conventional systemic chemotherapy lacks target selectivity, leading to high recurrence risks, multidrug resistance, and severe side effects. Transcatheter arterial chemoembolization (TACE) selectively blocks tumor blood supply to induce tumor cell necrosis through ischemia and hypoxia (Chang et al., 2021). Local delivery of chemotherapy drugs also enhances their anti-tumor effects (Qin et al., 2022). The combination of TACE with portal embolization or portal chemoembolization in patients with HCC improves efficacy and reduces recurrence rates (Shao et al., 2021). Although TACE is the preferred method of palliative care for HCC, the choice of a chemotherapy agent and the timing between administrations require further study (Ando et al., 2021). For HCC that cannot be cured, a liver transplant is the best way to restore liver function (Reichman et al., 2019). However, the shortage of liver transplant donors, immune rejection after liver transplantation, and prevention and treatment of liver cancer recurrence and metastasis after liver transplantation are still the direction of unremitting efforts in the future (Fan et al., 2023).

**SCHEME 1:**
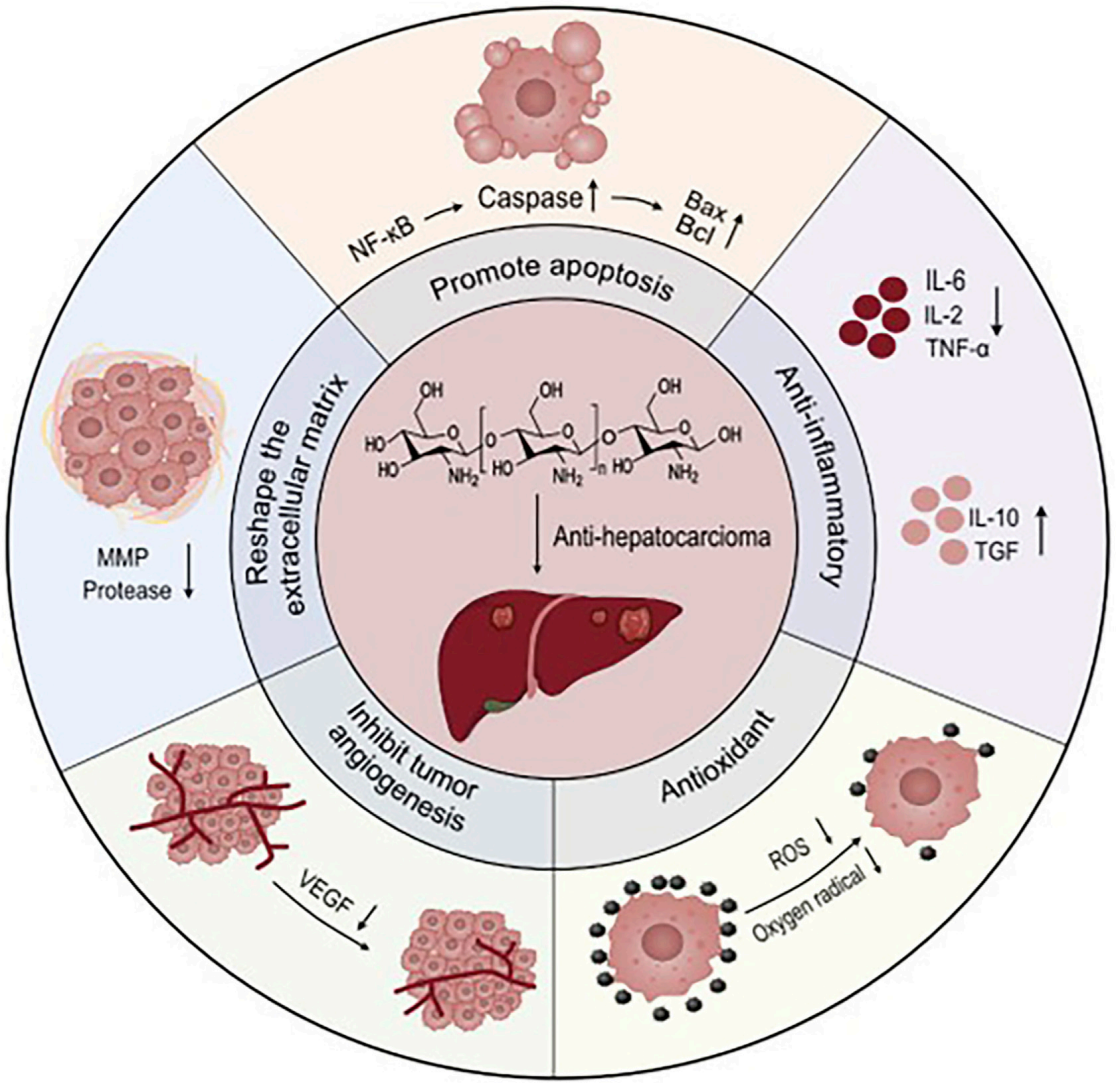
Mechanism of action of chitosan in hepatocellular carcinoma: Chitosan-based materials induce apoptosis of HCC cells by promoting mitochondrial pathway, promote downregulation of expression of anti-inflammatory cytokines, and upregulation of expression of inflammatory cytokines, promote ROS degradation, scavenge oxygen radicals around HCC cells. Reducing tumor angiogenesis, reduce MMP and protease expression and remodel the tumor extracellular matrix to achieve anti-tumor effects.

In recent years, nano-delivery strategies have shown promise in enhancing anti-tumor effects and have become a potential trend in cancer treatment. Chitosan, a biopolymer derived from chitin, is non-toxic and exhibits good histocompatibility and biodegradability (Mallakuntla et al., 2021; Abourehab et al., 2022; Saran et al., 2022; Kim et al., 2023; Matloob et al., 2023; Unal et al., 2023; El-Araby et al., 2024). It has been extensively studied as a delivery strategy for various tumors, including lung cancer, breast cancer, pancreatic cancer, and HCC (Xia Y. et al., 2022; Bashir et al., 2022; Karimi et al., 2023). Chitosan-based nanomaterials have shown significant anti-tumor effects, leveraging their ability to selectively enter cancer cells through the enhanced permeability and retention (EPR) effect (Xin et al., 2017). This article provides an overview of the physicochemical properties of chitosan. It also summarizes the anti-tumor effects of chitosan-based nanomaterials in HCC, as highlighted in Table 1; Scheme 1. Additionally, the challenges associated with the use of chitosan nanomaterials in HCC treatment are discussed.

**TABLE 1:** Chitosan-enhanced antitumor therapy for hepatocellular carcinoma.

Function	Name	Main composition	Material properties	Results	References
Induce apoptosis	Chitosan core-shell nanoparticles	Carboxymethyl chitosan, lactobionic acid, glycyrrhetinic acid and doxorubicin	The core-shell nanoparticles have a diameter of 274 nm	Chitosan nucleoshell nanoparticles selectively deliver chemotherapy drugs to liver tumors, inducing apoptosis of tumor cells	Hefnawy et al. (2020)
	DOX- TPP-CS NPs	Triphenylphosphine, chitosan, doxorubicin	DOX-TPP-CS NPS have a particle size of 70–110 nm	DOX-TPP-CS NPS effectively target DOX to liver tumor mitochondria to induce apoptosis	Arafa et al. (2022)
	DOX-Fe3O4@CGA	Graphene, chitosan, doxorubicin	The encapsulation efficiency of DOX is approximately 85%	DOX-Fe3O4@CGA shows strong synergistic oncology therapeutic potential	Chen et al. (2022a)
	GC-TP-NPs	Twtolide lactone, galactosylated chitosan	The particle size of GC-TP-NPs is 204.2 ± 1.2 nm	GC-TP-NPs induce apoptosis in HCC cancer cells by blocking TNF/NF-κB/BCL2 signaling	Zhang et al. (2019a)
	Chitosan nanoliposomes	Niacin, curcumin, chitosan	The particle size of Chitosan nanoliposomes is 96 ± 1.2 μm	Chitosan liposomes (the chitosan liposome with curcumin) can induce autophagy by activating the GPR109A/AMPK/NRF-2 signaling pathway	Hanafy et al. (2023)
Antioxidant	CS-5FU-CeO2 NPs	5-fluorouracil, chitosan, cerium oxide	The drug loading rate of CS-5FU-CeO2NPs was 16.17% ± 0.55%	CS-5FU-CeO2NPs synergistically enhance the anticancer activity of HepG2 cells by regulating ROS.	Sathiyaseelan et al. (2022)
	phosphorylated galactosylated chitosan (PGC)	5-Fluorouracil, galactosylated chitosan, cerium oxide	The average particle size of PGC is 197 nm	PGC can inhibit lipid peroxidation and superoxide scavenging ability and enhance glutathione levels	U et al. (2022)
	Chitosan co-encapsulation to make cur-cumin nanoparticles (CSCNP)	Chitosan, curcumin, 5-fluorouracil	The average size of CSCNP is 75.0 ± 14.62 nm	Compared to curcumin, CSCNP has a stronger oxidant free radical scavenging effect	Kong et al. (2019)
	Cela/GCTR PMs	Celastrol, glycyrrhetinic acid (GA) and carboxymethyl chitosan	The particle size of Cela/GCTR PMs is 220.17 ± 5.50 nm	Cela/GCTR PMs can target ROS in HCC cells to achieve antioxidant effects *in vivo*	Zhang et al. (2023)
	SF-CS NPs	chitosan, SF, tripolyphosphate	The largest spherical particles with an average diameter of 212.4 ± 59.7 nm	SF-CS NPs could continuously release SF for 169 h	Albalawi et al. (2023)
	Galactosylated chitosan nanoparticles	Chitosan, gemcitabine	The zeta potential values (19–22 mV) of galactosylated chitosan nanoparticles	The accumulation of galactosylated chitosan nanoparticles in the liver is significantly higher than that of other organs	Nair et al. (2019)
Anti-inflammatory	Chitosan-coated liposomes	Butyric acid, chitosan	The average chitosan liposome size of encapsulated BA is 126 nm	Chitosan-coated liposomes have important anti-inflammatory effects by inhibiting the production of cytokines	Quagliariello et al. (2019)
	5-FACN	Chitosan, aspirin and 5‐fluororacil	The average particle size of 5-FACN is 109.2 ± 5.2 nm	5-FACN is able to reduce COX-2 and prostaglandin expression around tumors	Wang et al. (2020)
	CMCS/SF-CLN	Sorafenib, carboxymethyl chitosan, lipids	The load ratio of CMCS/SF-CLN to SF is 7.43% ± 0.51%	CMCS/SF-CLN reduces TGF-β1 and IL-10 secreted by M2-TAM and M2-TAM	Wang et al. (2019b)
Inhibits tumor angiogenesis	CAN	Chitosan, polyacrylic acid and Rutin	The diameter of CAN is 116.7 nm	CAN reduces the expression of VEGF and inhibits tumor vascular formation	Radwan and Ali (2021)
	TLM-LCH NPs	Telmisartan, lactose-modified chitosan	TLM-LCH NPs have a diameter of 145.46 ± 0.7 nm	The TLM-LCH NPs group significantly reduced the expression levels of VEGF and MMP-2	Nasr et al. (2023)
	CMCS nanoparticles	Tim-3 siRNA, SF and CMCS	CMCS nanoparticles have a diameter of 50.49 ± 5.34 nm	CMCS nanoparticles induce a 95% reduction in tumor vascular density	Song et al. (2022)
	CMCS nanoparticles	CMCS, VEGF-siRNA and SF	—	VEGF-siRNA can target to lower VEGF around HCC cells, reduce tumor vascular production and induce early apoptosis	Yao et al. (2019)
	CS-SS-9R NPs	Nonaarginine, chitosan, VEGF-siRNA	—	VEGF expression decreased by 78.9%, tumor cell proliferation inhibited by 81.2%	Xu et al. (2018)
Promotes extracellular matrix remodeling	Chitosan-chondroitin nanoparticles	Chitosan, chondroitin, 5-FU	Chitosan-chondroitin nanoparticles have a particle size of 244.7 ± 16.3 nm	Chitosan-chondroitin nanoparticles can delay the degradation of ECM	Varshosaz et al. (2020)
	Chitosan nanoparticles	Apigenin, chitosan	Chitosan nanoparticles have a particle size of 189 nm	The Apigenin released by chitosan nanoparticles can downregulate the expression level of MMP-9 and delay HCC cell transfer	Mabrouk Zayed et al. (2022)

## 2 Physical and chemical properties of chitosan

Chitosan is a natural polysaccharide obtained by deacetylation of chitin (Xia Y. L. et al., 2022). When chitinin deacetylation reaches at least 50%, it is called chitosan (Hallmann and Gerngroß, 2022). The main component of chitosan is a mixture between N-acetyl-d-glucosamine and β-(1,4)-linked-d-glucosamine (Satitsri and Muanprasat, 2020). Due to its amino groups in the backbone, chitosan often exhibits positively charged cationic copolymers. Chitosan itself has been reported to have antitumor, antioxidant, and wound-healing effects (Manna et al., 2023). Chitosan is soluble in organic acids but insoluble in neutral and alkaline solutions. The solubility of chitosan depends on the amount of free amino group and N-acetyl group (Hallmann and Gerngroß, 2022). The physicochemical properties of CS are inversely affected by its degree of deacetylation and molecular weight (Ardean et al., 2021). Chitosan has active hydroxyl, amino, and linear polyamines at positions C2, C3, and C6, and these functional groups can be modified to confer different functions on chitosan (Balagangadharan et al., 2017). For example, the conversion of primary amine groups on chitosan C2 into quaternary salts is the main mechanism for enhancing its antibacterial, antioxidant, anticoagulant, and mucus adhesion properties (Piras et al., 2019). Therefore, chitosan plays an important role in tissue engineering and regenerative medicine. In tumor treatment, chitosan can target the tumor microenvironment, increase the Enhanced permeability and retention (EPR) effect, improve the delivery ability of anti-tumor drugs, and reduce the off-target and abscopal effect of cancer nano drugs (Xia Y. et al., 2022; Zaiki et al., 2023). Chitosan can also reduce cell proliferation, eliminate tumor angiogenesis, and inhibit HCC growth (Satitsri and Muanprasat, 2020). Chitosan-based nanostructures can characterize the pharmacokinetics of natural and synthetic drugs, thereby increasing the effectiveness of HCC therapy (Karimi et al., 2023). Nanomedicine developed using functionalized chitosan becomes a potential trend in tumor treatment.

## 3 Chitosan-based materials enhance antitumor therapy

### 3.1 Promote apoptosis of tumor cells

**FIGURE 1:**
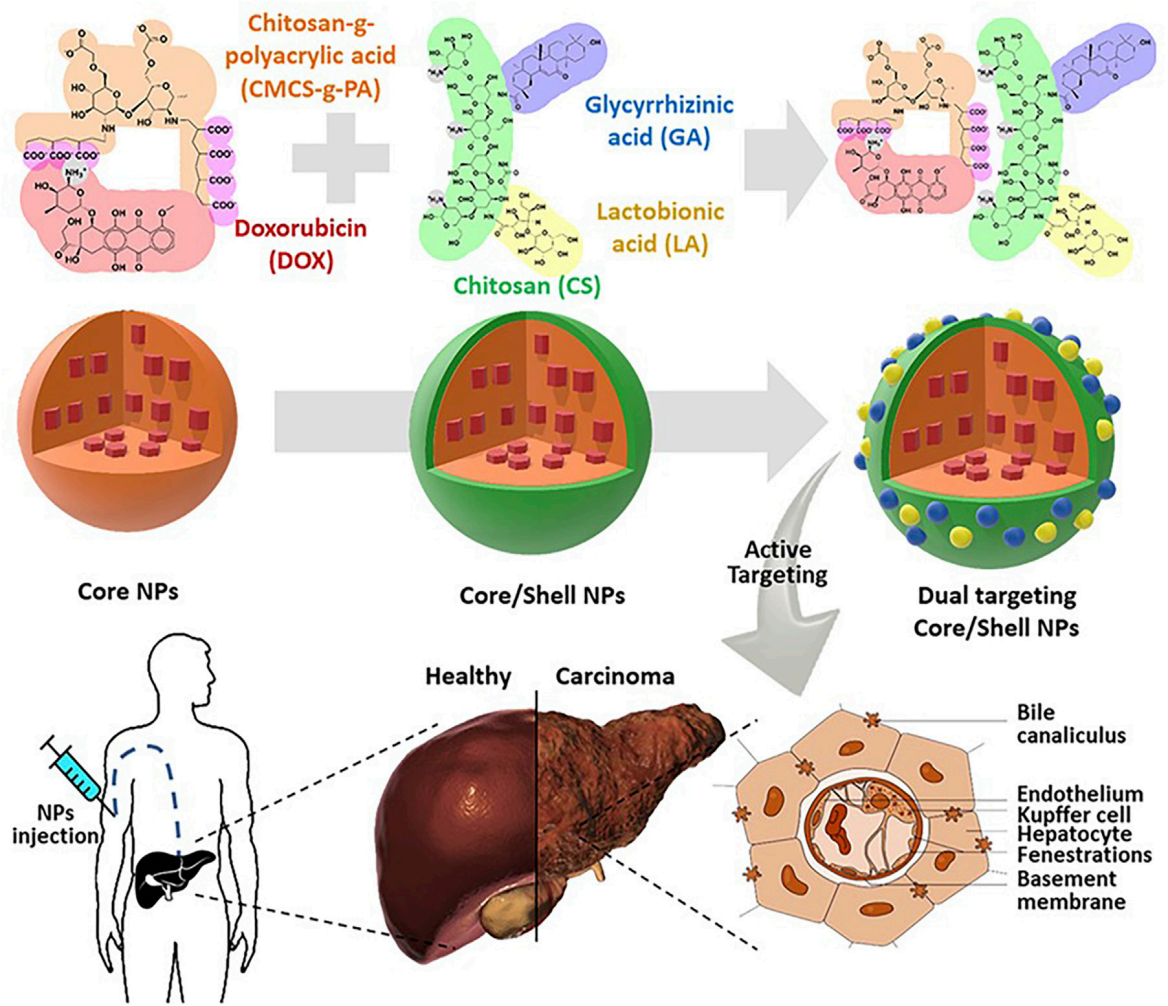
Carboxymethyl chitosan to complexe polyacrylate, and glycyrrhetinic acid (GA) and LA grafted onto the complex to make a two-ligand nanoshell structure for the delivery of DOX. After intravenous injection of nanoparticles, it can actively target liver cancer cells to achieve anti-tumor effects. Reproduced with permission from (Hefnawy et al., 2020).

Apoptosis is triggered by a series of mitochondria dysfunction, including the collapse of the intimal potential, swelling of the mitochondria, and increased permeability (Chipuk et al., 2006). Mitochondria control a variety of cellular physiological processes, including cell respiration, metabolism, signaling, differentiation, apoptosis, and intracellular calcium levels (Smith et al., 2011). Chitosan can induce apoptosis. The specific mechanism is that chitosan competitively blocks the integral proteins on tumor cells, so that the tumor loses the ability to adhere to normal tissues, thereby inhibiting tumor metastasis, and it can also directly enter the cell and activate the caspase-3 at the end of the apoptosis pathway, thereby causing the degradation of structural proteins and functional proteins, and finally disintegrating the cell (Atmaca et al., 2024). Mitochondrial function is associated with anabolic, unlimited multiplication and decreased apoptosis autophagy in cancer cells (Wang et al., 2017). Doxorubicin (DOX) is a mitotically active cyto-toxic agent that binds specifically to phospholipid cardiolipin and could accumulate mitochondria. Studies have shown that DOX-mediated membrane perturbation can inhibit mitochondrial membrane potential disruption of complex I and II disordered electron transport chains, thereby affecting cellular energy transfer (Gorini et al., 2018). Lactobionic (LA) has HCC cell targeting by binding to Asiatica protein (ASGP) receptors that are overexpressed on the HCC cell surface (Zhang et al., 2011). The grafting of LA and chitosan can enhance the HCC cell targeting ability of chitosan. Hefnawy et al. used carboxymethyl chitosan to complexe poly-acrylate, and glycyrrhetinic acid (GA) and LA grafted onto the complex to make a two-ligand nanoshell structure for the delivery of DOX (Figure 1) (Hefnawy et al., 2020). GA and LA double ligands enhance the HCC cell targeting ability of core-shell nanoparticles, and precisely release DOX into tumor cells to achieve their anti-tumor effects (Li et al., 2019). Studies have shown that nanoparticles larger than 150 nm are able to maximize the benefit of EPR effects in HCC cells (Torchilin, 2011). The core-shell nanoparticles have a diameter of 274 nm and are capable of loading DOX for more than 10 days. The ability of HCC cells to phagocytose DOX is greatly improved, and the significantly increased apoptotic genes are caspase 3, p53, and Bax. Triphenylphosphine (TPP) is one of the polymers commonly used to target mitochondria, and TPP has a unique structure composed of lipophilic phenyl groups and phosphine cations, which allows it to be deposited in mitochondria (Mossalam et al., 2010). Arisifa et al. grafted TPP with chitosan to deliver DOX to make DOX-TPP-CS NPS for the treatment of HCC (Arafa et al., 2022). The particle size of DOX-TPP-CS NPS is 70–110 nm, and the spherical and positive surface charge structure of DOX-TPP-CS NPTs enhances mitochondrial uptake. DOX-TPP-CS NPS continuously releases DOX at 168 h and reduces systemic toxicity. The co-incubation of DOX-TPP-CS NPS with HCC cells for 48 h compared with the blank control group promoted the programmed death of HCC cells by 7.86 times higher than that of the control group.

Coated magnetic Fe3O4 can absorb more drug molecules, increasing the dispersion and stability of chitosan nanoparticles (He et al., 2010). Coated chitosan nanoparticles can enhance the drug loading rate of chitosan nanoparticles, making chitosan nanoparticles a good choice for nano drug loading (Jędrzak et al., 2020). Chen L. et al. (2022) used Fe3O4-coated chitosan, DOX was loaded into CS by the aldehyde group, and GQD was prepared into DOX-Fe3O4@CGA on the surface of magnetic CS by amide bonds. The encapsulation efficiency of DOX is about 85%, and the loading efficiency is about 12% on average. The DOX-Fe3O4@CGA is injected into tumor-bearing mice and collected at the tumor site for 6 h to treat the tumor more effectively. TwHFOLIDE (TP) is the main component of Chinese herbal medicine and has anti-inflammatory and antitumor effects (Gali-Muhtasib et al., 2015). TP has been shown to have anti-tumor effects in hematologic tumors, lung cancer, liver cancer (Zhao et al., 2020; Gao et al., 2021). TP limits its clinical application due to its high toxicity and low water solubility. Galactosylated chitosan also has the ability to target HCC cells, binding to TP to deliver TP to tumor cells (Yu et al., 2014). Zhang Y-Q. et al. (2019) combined galactosylated chitosan with TP to make GC-TP-NPTs to enhance the targeting of HCC cells. The particle size of GC-TP-NPs was 204.2 ± 1.2 nm, GC-TP-NPs released about 70% TP within 2 h, and GC-TP-NPs released nearly 80.0% TP after 24 h of incubation. The precise targeting of GC-TP-NPs not only reduced hepatic and renal toxicity but also induced apoptosis of HCC cells through TNF/NF-κB/BCL2 signaling. Niacin can increase the NAD+/NADH ratio in the body and promote autophagy in tumor cells by inducing NAD+/NADH balance (Song et al., 2013). Hanafy et al. (2023) developed chitosan liposomes loaded with niacin and curcumin to achieve autophagy in HCC cells. The diameter of the nanoliposomes is 96 ± 1.2 μm. *In vivo*, chitosan liposomes can induce autophagy by activating the GPR109A/AMPK/NRF-2 signaling pathway. Sorafenib (SF) is an oral multi-kinase inhibitor with significant anticancer effects through antiproliferative, antiangiogenic and pro-apoptotic mechanisms (Juaid et al., 2021). Fahad Albalawi et al. fabricated chitosan nanoparticles (SF-CS NPs) for SF delivery using CS and sodium tripolyphosphate (TPP) (Albalawi et al., 2023). At the lowest concentration of TPP of 2.5 mg/mL, SF-CS NPs showed the largest spherical particles with an average diameter of 212.4 ± 59.7 nm. *In vitro* experiments confirmed that SF-CS NPs could continuously release SF for 169 h. Gemcitabine is a pyrimidine nucleoside antimetabolite that is effective in the treatment of HCC in combination with other anticancer drugs, including sorafenib, oxaliplatin, carboplatin, and bevacizumab (Fan et al., 2023). However, adverse effects associated with gemcitabine and myelosuppression and pulmonary toxicity remain a problem (Chi et al., 2012). Nair et al. (2019) designed galactosylated chitosan nanoparticles for delivery of gemcitabine to reduce its toxic side effects. The zeta potential values (19–22 mV) of galactosylated chitosan nanoparticles increase modest rejection and electrostatic stabilization between galactosylated chitosan nanoparticles to provide stability. *In vivo* experiments have proved that galactosylated chitosan nanoparticles can release about 85% of gemcitabine within 24 h, and the drug distribution *in vivo* has proved that the accumulation of galactosylated chitosan nanoparticles in the liver is significantly higher than that of other organs, reducing the adverse reactions in the whole body. The study of chitosan-based nanomaterials to promote apoptosis in HCC cells has been widely confirmed (Priya et al., 2020).

### 3.2 Antioxidant

After mitochondrial dysfunction, tumor cells are in a hypoxic state. Long-term hypoxia leads to enhanced glycolytic pathways, enhanced hypoxia-inducible factor (HIF-1α), and the production of Reactive oxygen species (ROS) (Zhang W. et al., 2019). The imbalance between ROS and antioxidants in the body is one of the factors that promote the proliferation of tumor cells (Snezhkina et al., 2019). Mitochondria are the main organs for ROS production, and most anti-cancer drugs alter excess ROS production in cancer cells, thereby activating mitochondrial intrinsic pathways, releasing pro-apoptotic factors, and leading to apoptosis (Perillo et al., 2020). Cerium oxide (CeO2) exerts antioxidant activity by removing ROS from the body (Siposova et al., 2022). Sathiyaseelan et al. (2022) used CeO2 modified 5-fluorouracil (5FU)-loaded chitosan nanoparticles to make CS-5FU-CeO2NPs for HCC cell therapy. 5FU binds to chitosan through hydrogen bonding and intermolecular force, which greatly increases the drug loading rate of CS-5FU-CeO2NPs (16.17% ± 0.55%). *In vitro* experiments, CS-5FU-CeO2NPs released 21.88% of 5-FU within 8 h and sustained release within 6 months. Compared with CeO2NPs, CS-5FU-CeO2NPs greatly improved the scavenging capacity of free radicals and promoted HCC apoptosis (apoptotic cell mortality was 26.04%). Although mitochondria are the main site of ROS production, CS-5FU-CeO2NPs clearance of ROS does not cause damage to cellular structures (nuclear and mitochondrial membrane potentials). This also makes CS-5FU-CeO2NPs have good biocompatibility and low toxicity. Scavenging of oxidative free radicals in the body can promote apoptosis of tumor cells. Targeted induction of ROS production also induces apoptosis in tumor cells. Chitosan nanoparticles can induce increased ROS production, leading to ROS-induced activation of mitochondrial disease and endoplasmic reticulum stress (Jiang et al., 2019). Anushree et al. developed phosphorylated galactosylated chitosan (PGC) for antioxidant use in HCC cells (U et al., 2022). The average particle size of PGC is 197 nm, and PGC has a high affinity with ASGPR on the surface of HCC cells, which can enhance the HCC cell targeting ability of PGC (Zhu et al., 2022). *In vivo* experiments have confirmed that PGC can inhibit lipid peroxidation and superoxide scavenging ability and enhance glutathione levels compared with chitosan. PGC exhibits stronger anti-tumor effects than chitosan.

Curcumin is a polyphenolic compound whose clinical effects have been widely proven, including anti-inflammatory, antioxidant, antitumor, antiviral, antibacterial and analgesic effects (Hewlings and Kalman, 2017). Studies have shown that curcumin’s anti-inflammatory properties are achieved by blocking IκBα phosphorylation and degradation (Wilken et al., 2011). In addition to this, curcumin scavenges superoxide, nitric oxide, and hydrogen peroxide free radicals and reduces inflammation by lowering histamine levels (Alok et al., 2015). Due to low water solubility, low bioavailability, chemical instability, rapid metabolism in the gastrointestinal tract, and other factors, the clinical application of curcumin is limited. Kong et al. (2019) used silica encapsulation curcumin nanoparticles (SCNP) and chitosan co-encapsulation to make cur-cumin nanoparticles (CSCNP). The average size of CSCNP is 75.0 ± 14.62 nm and capable of loading 28.9% curcumin. *In vitro* experiments have confirmed that CSCNP does not affect the physiological activity of normal cells. Compared to curcumin, CSCNP has a stronger oxidant free radical scavenging effect. Celastrol is a natural proteasome inhibitor extracted from Chinese herbal medicine, according to a wide range of antitumor effects (Wang et al., 2019). However, the low solubility, low bioavailability and systemic toxicity of Celastrol hinder its clinical application (Chen et al., 2022). Zhang et al. (2023) used glycyrrhetinic acid (GA) and carboxymethylchitosan (CMCS) to make polymer micelles Cela/GCTR PMs for the delivery of Celastrol. HCC cells showed high expression of GA receptors, and GC-made Cela/GCTR PMs have good HCC cell targeting, thereby increasing HCC cell accumulation in Celastrol. The particle size of Cela/GCTR PMs is 220.17 ± 5.50 nm. Cela/GCTR PMs release Celastrol continuously, with a cumulative release rate of >12% at 70 h. Studies have shown that Cela/GCTR PMs can target ROS in HCC cells to achieve antioxidant effects *in vivo*. Compared with Celastrol, Cela/GCTR PMs showed good proliferation inhibition in hepatoma cells. Not only that, Cela/GCTR PMs have a stronger tumor suppression rate. This shows that Cela/GCTR PMs have great potential as an anti-liver cancer drug delivery system.

### 3.3 Anti-inflammatory

Inflammation is one of the important features of tumor-contributing markers and plays an important role in tumorigenesis (Hanahan, 2022). Inflammation can regulate and induce cell polarization in the tumor microenvironment and induce the development of tumor cell drug resistance (Denk and Greten, 2022; Kennel et al., 2023). Factors released by inflammatory cells (transforming growth factor (TGF)-β, tumor necrosis factor (TNF)-α, and interleukin (IL)-6) stimulate tumor cell survival and proliferation through nuclear factor (NF)-κB and signal transductors and transcriptional activators (STAT) 3 (Greten and Grivennikov, 2019; Kruse et al., 2023). M2-macrophages, fibroblasts, and myeloid-derived suppressor cells can induce immunosuppressive blocker antitumor effects of T cells (Kuo et al., 2022). Extended activation of the IL-6/IL-6-R signaling pathway is critical in the occurrence and progression of HCC (Hatting et al., 2015). Therefore, reducing the inflammatory response in the tumor microenvironment has a degree of antitumor effect. Butyric acid (BA) reduces the production of cytokines (IL-6, IL-8, TNF-α, and TGF-β) to achieve anti-inflammatory effects in HCC (Meijer et al., 2010). In addition, BA shows anticancer properties against HCC cells mainly based on its histone deacetylase (HDAC) inhibitory activity (Coradini and Speranza, 2005). However, the low bioavailability of BA and poor intestinal absorption after oral administration limit its clinical use (Clemente et al., 2012). Quagliariello et al. (2019) prepared chitosan liposomes, which were legally loaded with BA by membrane water for BA delivery. The chitosan liposomes that encapsulate BA have an average size of 126 nm and can continuously release BA around tumor cells. Chitosan liposomes that encapsulate BA have good cytocompatibility and do not cause toxicity to normal cells. *In vitro*, experimental results showed that chitosan liposomes encapsulated BA could reduce the production of IL-8, IL-6, TGF-β, and TNF-α to achieve anti-inflammatory effects.

Aspirin is a commonly used nonsteroidal anti-inflammatory drug in clinical practice, and the most well-known biological target of aspirin is cyclooxygenase 2 (COX-2) (Menter and Bresalier, 2023). COX-2 is highly expressed in HCC to convert arachidonic acid to prostaglandins, thereby helping to promote HCC cell proliferation and inhibit apoptosis (Kern et al., 2004). Aspirin inhibits cell migration and induces apoptosis in human HCC cells by inhibiting the activation of the NF-κB pathway, downregulating COX-2 levels (Dong et al., 2014). A meta-analysis by Zeng et al. (2023) has confirmed that aspirin use is associated with a reduced risk of hepatocellular carcinoma. Wang et al. (2020) developed a chitosan nanoparticle for the delivery of aspirin and 5‐fluororacil (5-FACN). The average particle size of 5-FACN is 109.2 ± 5.2 nm. The encapsulation efficiency of 5-FACN for 5-Fu and aspirin was 88.6% and 91.0%, respectively. 5-FACN can continuously release 5-Fu and aspirin around tumor cells. Studies have shown that 5-FACN is able to reduce COX-2 and prostaglandin expression around tumors. Macrophages are involved in all stages of tumor progression and are associated with poor prognosis and chemotherapy resistance (Bohn et al., 2018). Tumor-associated macrophages (TAMs) are mainly divided into anti-tumor M1-TAM and protumor M2-TAM (Sica et al., 2008). In tumor progression, the pro-tumor function of TAM is caused by NF-κB activation (Li et al., 2017). It has been reported that NF-κB can promote the polarization of TAM from M1 to M2, and inhibiting NF-κB activation can enhance the ratio of M1/M2 (Taniguchi and Karin, 2018). Fan et al. (2021) designed chitosan nanopole capsules for the delivery of cisplatin, named PC-CP. PC-CP can cause less fibroblast response and less macrophage response in the tumor microenvironment.

Wang T. et al. (2019) prepared cationic lipid-based nanoparticles (SF-CLN) loaded with SF, and coated CMCS in SF-CLN to prepare CMCS/SF-CLN for targeting HCC cells. The load ratio of CMCS/SF-CLN to SF is 7.43% ± 0.51%. CMCS with a negative charge can make CMCS/SF-CLN repel from normal cell membranes and reduce the cytotoxicity of CMCS/SF-CLN. In HCC cells, CMCS/SF-CLN has charge-inversion properties and can adapt to the acidic environment of tumor cells and aggregate in large quantities. *In vivo*, experiments confirmed that CMCS/SF-CLN has a good ability to target HCC and reduce TGF-β1 and IL-10 secreted by M2-TAM and M2-TAM. Compared with SF, CMCS/SF-CLN has better anti-inflammatory and anti-tumor effects.

### 3.4 Inhibits tumor angiogenesis

Tumor angiogenesis plays an important role in the growth and metastasis of tumor cells (Park et al., 2007). Tumor blood vessels provide nutrients and oxygen to tumor cells and carry away metabolic waste products produced by tumor cell metabolism (Miura et al., 2010). The formation of new blood vessels is the result of the intermodulation of proangiogenic compounds such as vascular endothelial growth factor (VEGF), transforming growth factor-β (TGF-β), basic fibroblast growth factor (bFGF), matrix metalloproteinases (MMP), platelet-derived growth factor (PDGF), and antiangiogenic factors such as tissue inhibitors of angiostatin, endostatin, and metalloproteinases (Zhou et al., 2022). Inhibition of tumor angiogenesis inhibits tumor cell proliferation. CMCS has been shown to inhibit tumor angiogenesis and downregulate levels of VEGF and TIMP-1, inhibitors of MMP (Jiang et al., 2015). Rutin has antioxidant, anti-inflammatory, antithrombotic and cytoprotective activities (Ma et al., 2018). However, Rutin’s poor water solubility and low bioavailability limit its clinical use. Radwan and Ali (2021) used chitosan and poly (acrylic) to make nano gels (CANs) for the delivery of Rutin. CAN has a diameter of 116.7 nm and is connected to Rutin by hydrogen bonding. CAN release Rutin around HCC cells, upsetting the balance of angiogenesis and disruption. Compared to Rutin, CAN reduced the expression of VEGF and inhibited tumor vascular formation. Not only that, CAN also reduces the proliferation of HCC cells and promotes HCC apoptosis.

**FIGURE 2:**
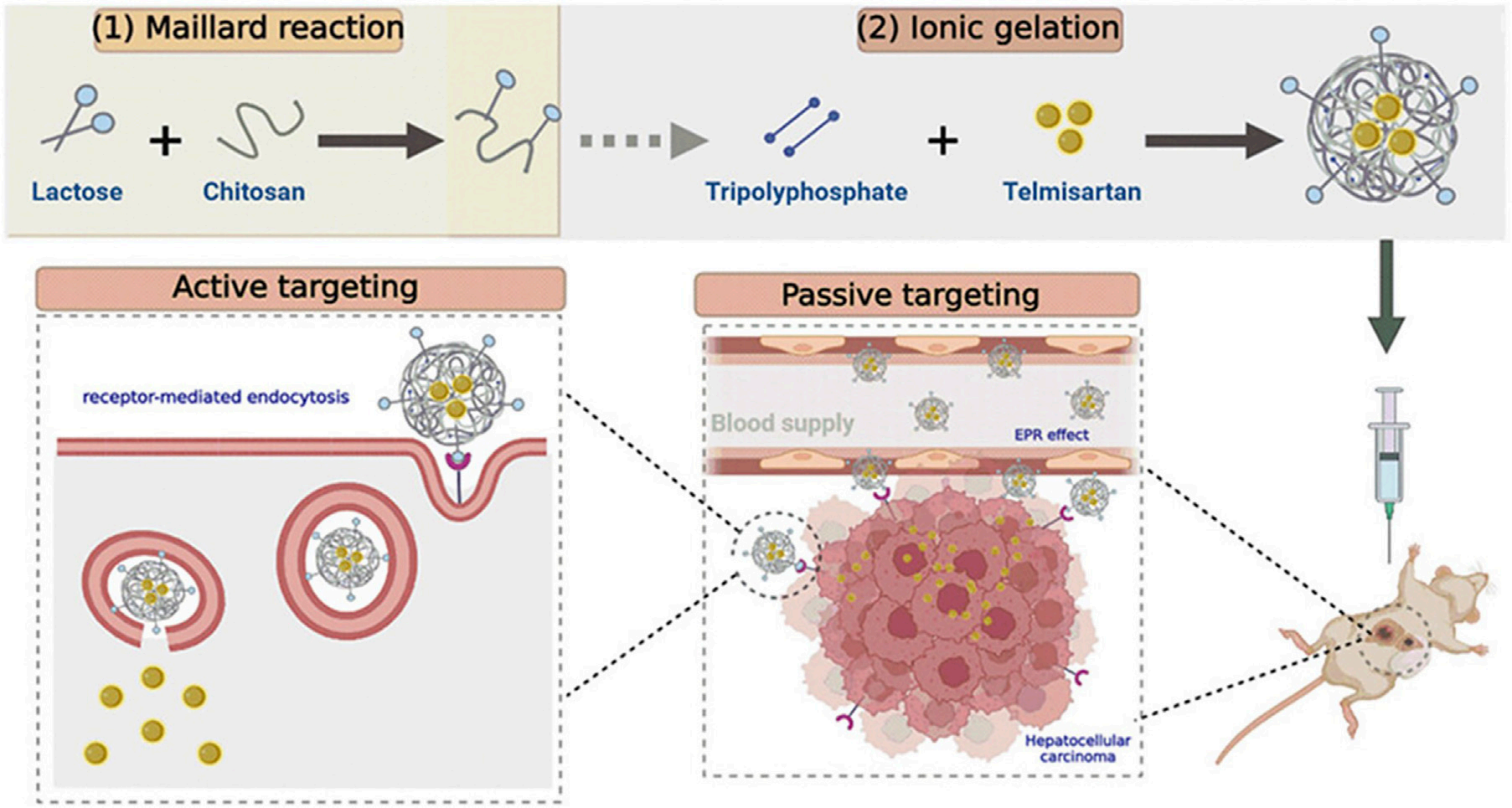
Lactose-modified chitosan nanoparticles (LCH NPs) were used as a delivery system for the delivery of telmisartan. After intravenous injection of nanoparticles, the nanoparticles can enhance the targeting effect on the liver, release telmisartan, and enhance its anti-tumor effect. Reproduced with permission from (Nasr et al., 2023).

Telmisartan (TLM) alleviates malignant cell proliferation by activating peroxisome proliferator-activated receptor γ (Li et al., 2014). Nasr et al. (2023) designed lactose-modified chitosan nanoparticles to deliver TLM (TLM-LCH NPTs) to enhance the uptake of TLM by HCC cells (Figure 2 (1, 2)). TLM-LCH NPs have a diameter of 145.46 ± 0.7 nm. Studies have confirmed that lactose-modified chitosan nanoparticles actively target ASGPR and enhance the up-take of nanoparticles by HCC cells. TLM-LCH NPs released TLM aggregates in HCC cells at a content 232.92 times higher than that of TLM alone. Compared with the common TLM group, the TLM-LCH NPTs group significantly reduced the expression levels of VEGF and MMP-2. Not only that, TLM-LCH NPs also reduce inflammation around tumor cells and reduce the expression level of alpha-fetoprotein. T cell immunoglobulin mucin-3 (Tim-3) is a promising immune checkpoint molecule for HCC therapy (Romero, 2016). Inhibition of Tim-3 expression may be a novel therapeutic strategy for HCC. Song et al. (2022) grafted Tim-3 siRNA and SF to CMCS nanoparticles by a single emulsification method. Tim-3 siRNA can target and inhibit Tim-3 expression in HCC cells. The diameter of the nanoparticles is 50.49 ± 5.34 nm, and 90% of SF can be released *in vivo* for 40 h. *In vivo* experiments have shown that CMCS nanoparticles induce a 95% reduction in tumor vascular density and enhance the recruitment of cytotoxic T cells to kill tumor cells. In another study, Yao et al. (2019) used CMCS nanoparticles to deliver VEGF-siRNA and SF. VEGF-siRNA can target lower VEGF around HCC cells, reduce tumor vascular production, and induce early apoptosis. Xu et al. (2018) grafted nonaarginine (9R) onto chitosan (CS) and constructed a positively charged kernel (CS-SS-9R) for delivery of VEGF-siRNA. *In vivo* experiments confirmed that siVEGF was rapidly released into the cytoplasm, resulting in a 78.9% decrease in VEGF expression and 81.2% inhibition of tumor cell proliferation.

### 3.5 Extracellular matrix remodeling

The extracellular matrix (ECM) is composed of proteins, glycoproteins, and polysaccharides and is an important component of the tumor microenvironment (TME) (Lu et al., 2012). The ECM is a dynamically changing system that provides nutrients to the tumor cell parenchyma and regulates tumor cell growth and metabolism (Bissell and Hines, 2011). ECM is an important factor for tumor cells to escape attack by the immune system (Roy et al., 2023). Chronic inflammation and dysregulation of ECM remodeling work together to contribute to an immunosuppressive environment, which in turn promotes HCC proliferation, invasion, and metastasis (Chen et al., 2023). In addition, ECM, which also has a large number of immunosuppressive cells (regulatory T cells, myeloid-derived suppressor cells, tumor-associated fibroblasts, etc.) and cytokines (TGF-β, VEGF, or IL-10), promotes tumor cells to escape attack by the immune system (Chiang et al., 2008). Studies have shown that HCC cells are able to cause abnormal ECM deposition through the WNT/TGFB signaling pathway, leading to intratumor fibrosis (Desert et al., 2023). Liu et al. (2023) confirmed that HCC cells participate in the regulation of ECM through the PI3K/AKT signaling pathway, which leads to tumor cell proliferation, migration, and invasion. Xu et al. (2022) confirmed that MMP in ECM is associated with poor prognosis of HCC. Therefore, regulatory ECM remodeling is a potential clinical mechanism for the treatment of HCC.

**FIGURE 3:**
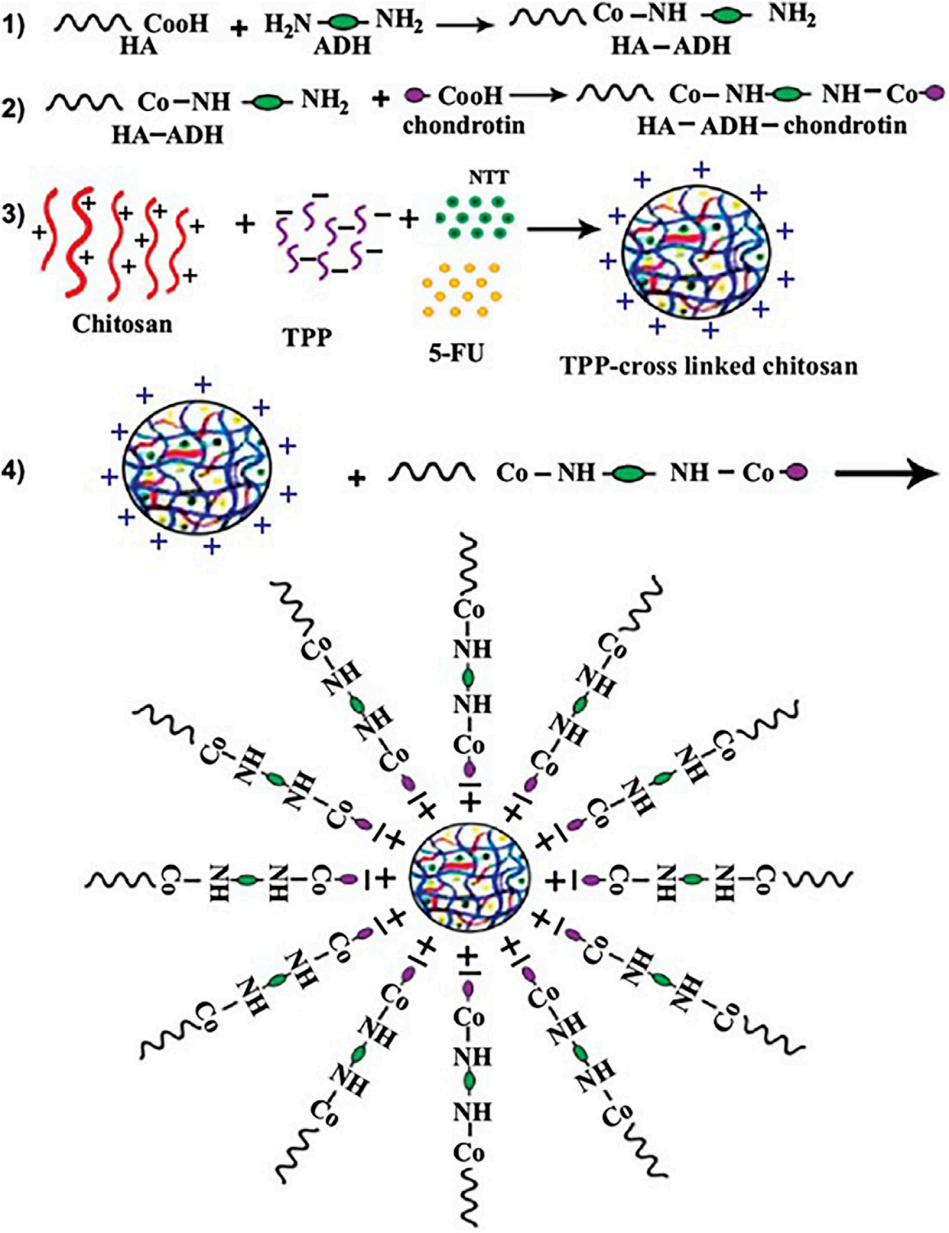
5-FU and Nitroxoline (NIT) were loaded in chitosan-chondroitin nanoparticles. To target the CD44 receptors of HepG2 cells, Hyaluronic Acid (HA) was conjugated to the chondroitin by adipic acid dihydrazide and the conjugation was confirmed by FTIR and 1HNMR. Target nanoparticles co-delivery of 5-FU and NIT to enhance the 5-FU cytotoxic effects and reduce the metastatic properties of HepG2 cells. Reproduced with permission from (Varshosaz et al., 2020).

Delaying the degradation of host proteins in ECM has advantages in inhibiting tumor cell proliferation (Lu et al., 2012). Nitrotrixine (NIT) is a potent inhibitor of cathepsin B that impairs tumor progression by reducing extracellular matrix degradation (Spottiswoode et al., 2023). Varshosaz et al. (2020) loaded 5-FU and NIT in chitosan-chondroitin nanoparticles (Figure 3 (1–4)). Chondroitin binds to hyaluronic acid in ECM to enhance the targeting of HCC cells by nanoparticles. The particle size of chitosan-chondroitin nanoparticles is 244.7 ± 16.3 nm, the loading rate of 5-FU is 3.5% ± 0.5%, and the loading rate of NIT is 75.1% ± 0.9%. Chitosan-chondroitin nanoparticles can continuously release the carrier, releasing about 6.0% ± 9.5% of 5-FU and 62.9% ± 0.7% of NIT at 8 h. *In vivo* experiments have shown that chitosan-chondroitin nanoparticles can delay the degradation of ECM and reduce the proliferation and migration of HCC cells compared with NIT. MMP9 plays an important role in proteolysis, membrane peptide degradation, and extracellular protein denaturation of extracellular matrices. This protein denaturation promotes cancer cell proliferation, which promotes metastasis (Deryugina and Quigley, 2006). Zayed et al. developed chitosan nanoparticles for the delivery of Apigenin (Mabrouk Zayed et al., 2022). Apigenin has powerful anticancer, anti-inflammatory and antioxidant activities (Yan et al., 2017). Chitosan nanoparticles have a particle size of 189 nm and are capable of continuous release of Apigenin *in vitro*, with a 40-h drug release rate of 24%. The Apigenin released by chitosan nanoparticles can downregulate the expression level of MMP-9 and delay HCC cell transfer.

## 4 Challenges for chitosan-based materials

Although chitosan-based biomaterials have achieved excellent results in tumor delivery, the clinical application of chitosan-based materials also presents certain challenges (Patel and Goyal, 2017). Chitosan and chitin are almost inseparable, and chitin, when the deacetylation of chitin reaches at least 50%, is called chitosan (Hallmann and Gerngroß, 2022). Chitin has more protein components than chitosan and therefore has a stronger ability to activate the immune system (Thambiliyagodage et al., 2023). However, too much protein makes chitosan enhance anti-tumor by inducing activation of the immune system, but also causes allergic symptoms in some patients (Taokaew and Kriangkrai, 2023). The purification of chitosan mainly includes strong acid, strong base and enzymatic degradation (Harish Prashanth and Tharanathan, 2007; Islam et al., 2022). Enzymatic degradation is extremely expensive, and large-scale production of high-purity chitosan is still a problem to be solved (Herdiana et al., 2023). Secondly, the biggest challenge of chitosan is its low solubility and poor mechanical properties (Szymańska and Winnicka, 2015). Chitosan is incompatible with hydrophobic chemotherapy drugs and, therefore, has limitations in chemotherapy drug delivery (Herdiana et al., 2023). This necessitates the modification of chitosan materials (Huang et al., 2023). Stability is another challenge affecting chitosan applications. There are significant differences in the effects of molecular weight, degree of acetylation, and purity level of chitosan materials on the stability of chitosan-based materials (Szymańska and Winnicka, 2015). Negatively charged components (gelatin, hyaluronic acid, alginate, etc.) are often crosslinked with chitosan to improve their stability (Hamman, 2010). Therefore, more preclinical studies are needed to improve the stability of chitosan-based materials.

## 5 Conclusion and outlook

Biomaterials drug delivery strategies to improve anti-tumor therapy have become a research hotspot in recent years. As a type of natural polysaccharide, chitosan has been shown to have antitumor activity due to its biological histocompatibility, low toxicity and positive charge. However, the anti-tumor properties of chitosan are not as significant as one might expect. Due to unfavorable factors such as low solubility, poor mechanical properties, low yield, and poor stability of chitosan, its clinical application is limited. There is an urgent clinical need for a bioactive material with low toxicity, target specificity and excellent drug delivery properties. To this end, a variety of improved chitosan-based biomaterials have been designed for anti-tumor therapy in preclinical studies. Studies have confirmed that chitosan-based biomaterials can promote mitochondria-induced apoptosis, promote tumor cell antioxidants, and reduce the production of IL-8, IL-6, TGF-β, and TNF-α to achieve anti-inflammatory effects. In addition, chitosan-based materials can enhance anti-tumor therapy by inhibiting the expression of VEGF to reduce tumor angiogenesis and promote extracellular matrix remodeling. However, the treatment of HCC by chitosan-based biomaterials is still only in preclinical studies and has not been reported clinically. Therefore, future research should focus on addressing the above difficulties to realize the full potential of chitosan-based biomaterials.
